# Peri-Operative Management of Older Adults with Cancer—The Roles of the Surgeon and Geriatrician

**DOI:** 10.3390/cancers7030853

**Published:** 2015-08-18

**Authors:** Ruth Mary Parks, Siri Rostoft, Nina Ommundsen, Kwok-Leung Cheung

**Affiliations:** 1 School of Medicine, University of Nottingham, Royal Derby Hospital Centre, Derby DE 22 3DT, UK; E-Mail: ruth.parks@nhs.net; 2 Department of Geriatric Medicine, Oslo University Hospital, Oslo 0424, Norway; E-Mail: srostoft@gmail.com; 3 Department of Geriatric Medicine, Akershus University Hospital, Po box 1000, Lørenskog 1478, Norway; E-Mail: ninaommundsen@gmail.com

**Keywords:** comprehensive geriatric assessment, cancer in the older adult, multidisciplinary team, geriatrician, surgeon

## Abstract

Optimal surgical management of older adults with cancer starts pre-operatively. The surgeon plays a key role in the appropriate selection of patients and procedures, optimisation of their functional status prior to surgery, and provision of more intensive care for those who are at high risk of post-operative complications. The literature, mainly based on retrospective, non-randomised studies, suggests that factors such as age, co-morbidities, pre-operative cognitive function and intensity of the surgical procedure all appear to contribute to the development of post-operative complications. Several studies have shown that a pre-operative geriatric assessment predicts post-operative mortality and morbidity as well as survival in older surgical cancer patients. Geriatricians are used to working in multidisciplinary teams that assess older patients and make individual treatment plans. However, the role of the geriatrician in the surgical oncology setting is not well established. A geriatrician could be a valuable contribution to the treatment team both in the pre-operative stage (patient assessment and pre-operative optimisation) and the post-operative stage (patient assessment and treatment of medical complications as well as discharge planning).

## 1. Introduction

Age is the single biggest risk factor for developing cancer and most cancer-related deaths occur in the older age group. It is well recognised that age alone should not determine treatment options [1] however, undertreatment in this age group is common [2,3,4]. Older patients are more likely to have malignant than benign tumours, therefore, surgery with curative intent becomes more necessary. The older patient is likely to have a greater number of comorbidities as well as functional and cognitive impairment, which may make decision making regarding surgery challenging. It has been shown that active cancer alone compared to patients with no active cancer increases risk of mortality within 30 days of non-cardiac surgery [5].

Tumours in the elderly may have different histological features. Alongside geriatric considerations as will be discussed below, it means that treatment offered may differ from that of their younger counterparts, with non-surgical options as potential alternatives (Table 1). Therefore, it is of paramount importance that all treatment options are considered in conjunction with the patient and multi-disciplinary team (MDT) and this should include surgery where appropriate.

**Table 1:** Comparison of surgery with potential non-operative treatment—notable examples.

Cancer	Surgical	Non-Surgical
Breast	Mastectomy or wide local excision	Primary endocrine therapy or radiotherapy
Prostate	Radical prostatectomy	Endocrine therapy or radiotherapy
Rectal	Rectal resection	Chemoradiotherapy
Lung (non small-cell)	Lobectomy or pneumonectomy	Radical radiotherapy

Geriatricians are used to evaluating and treating older patients with comorbidities, polypharmacy, disabilities, and cognitive impairment. One method of analysing this is by Comprehensive Geriatric Assessment (CGA), which is a “multidimensional and usually interdisciplinary diagnostic process designed to determine a frail older person’s medical conditions, mental health, functional capacity and social circumstances. The purpose is to plan and carry out a holistic plan for treatment, rehabilitation, support and long term follow up” [6]. Based on a geriatric assessment is it possible to identify older patients who are frail and thus at risk of surgical complications. Frailty is a term used as a marker of vulnerability, identifying individuals with a reduced capacity to respond to external stressors such as surgery [7]. It is linked to advanced age and disease-related processes, but it is generally accepted that frailty is a separate entity. If the older patient is frail and has a limited life expectancy combined with a high risk of surgical complications, the goals of treatment may deviate from what is expected in a younger or fitter patient. CGA can allow an individualised patient plan and treatment goal to be established. This opinion paper primarily focuses on the potential and emerging integrative roles of the surgeon and geriatrician in this environment.

## 2. Pre-Operative Management

### 2.1. Should We Operate?

A number of factors have deemed that discussion regarding treatment options is more complicated than in the past, including greater need for information by the patient, more effective utilisation of available resources, advances in peri-operative management and surgical techniques. Currently there is no “laboratory test” available to predict adverse post-operative outcome. The responsibility lies within the MDT to plan treatment in a systematic manner by considering treatment options as a whole (to treat or not to treat, and to treat with surgery or non-surgical methods) and then considering surgical treatment options (such as minimally invasive procedures) where appropriate. Patient related factors and optimisation of these should be considered throughout.

From a surgical perspective, the aim is to select the most appropriate surgical procedure and optimise functional status in order to prevent post-operative complications in these patients. Surgical priorities may be different for visceral *versus* non-visceral surgery. Patients undergoing visceral surgery are likely to benefit significantly more from minimally invasive surgery, prehabilitation and enhanced outcomes after surgery (ERAS) programmes, as will be described below.

### 2.2. Which Surgical Procedure?

The key predictors of surgical outcome include the following: (i) extent of surgical insult, (ii) duration of surgery; (iii) blood loss during surgery.

(i) Extent of Surgical Insult

Minimally invasive surgery is becoming more popular with surgeons and patients. Such techniques have been shown to have as good long term outcomes as well as fewer post-operative complications, when compared to open procedures in some cancers, for example, colorectal [8,9], lung [10] and upper gastrointestinal cancers [11].

The Colon Cancer Laparoscopic or Open Resection (COLOR) study [12] compared 627 patients randomly allocated to laparoscopic surgery with 621 allocated to open surgery for cancer of the right or left colon. Median age of participants was 71 years (range 27–95). The laparoscopic patients benefited from earlier recovery of bowel function, lower analgesia requirement and shorter length of hospital stay. Morbidity and mortality at 28 days was the same for both cohorts. The study concluded that laparoscopic surgery can be used for safe resection of cancer in the right, left and sigmoid colon.

A propensity matched analysis by Subroto *et al.* [10] compared long term survival of patients with lung cancer undergoing either open lobectomy or thoracoscopic surgery. A total of 1195 patients aged over 65 years, in each group were compared. There was no significant difference between the groups at three years in terms of overall survival, disease-free survival or cancer-specific survival.

Zhao *et al.* [11] analysed 63 consecutive patients with oesophageal cancer who either underwent minimally invasive or open transthoracic resection. Mean age of participants was 60 years (range 52–74). None of the patients had neoadjuvant therapies. There were no in-hospital mortalities in either group and both groups had comparable morbidity and oncological outcome.

These studies suggest that minimally invasive procedures have similar overall survival to that of open procedures and therefore should be considered particularly in the elderly patients with impaired functional status, with the aim to decrease the extent of surgical insult and therefore, post-operative complications, shorten length of stay and promote quicker recovery.

(ii) Duration of Surgery

Longer duration of surgery has been found to have adverse outcome on factors such as length of longer length of hospital stay, more postoperative complications and decreased mortality in major abdominal surgery [9], head and neck cancer surgery [13] and oesophageal cancer surgery [14] in the elderly population.

Although minimally invasive techniques such as those mentioned above may provide less surgical insult, they may require more time and expertise. The COLOR study [12] found that laparoscopic procedures lasted significantly longer than open procedures. This is echoed in the study by Zhao *et al.* [11] who again found statistically significant difference between patients undergoing the minimally invasive procedure. Therefore, in conjunction with the anaesthetist, risks and benefits of minimally invasive procedures *versus* anaesthesia/surgical time should be weighed up.

(iii) Blood Loss

Greater blood loss, measured as intraoperative blood loss and requirement for blood transfusion, has been shown to be independently associated with postoperative complications in oesophageal and gastric cancer surgery [14,15]. Less blood loss has been reported with laparoscopic procedures in colorectal surgery [16,17], gastric surgery [18] and cervical cancer surgery [19], in the elderly.

### 2.3. Prehabilitation

Once the decision is made to proceed to surgery and the surgical procedure has been determined, means of prehabilitation should be considered in all patients. Which patients will benefit from prehabilitation depends on the surgical procedure. Prehabilitation is the period of time from diagnosis to treatment in which functional capacity can be improved by a variety of means including: optimisation of comorbidities, improved nutritional status and cardiopulmonary function (See Table 2) [20,21]. Because functional disability is an important predictor of outcome, it is likely that prehabilitation could improve outcome in older surgical cancer patients.

This is a concept that has been utilised in the setting of orthopaedics [22,23] and cardiac surgery [24,25,26] to good effect. Its use in oncological surgery is evolving particularly in the field of colorectal cancer [21] and a number of clinical trials in this area are in progress [27].

A pilot study by Li *et al*. [28] examined consecutive patients with colorectal cancer over a 23-month period who were allocated to either a prehabilitation programme (N = 42) or no intervention (N = 45). The mean age of the prehabilitation group was 67.4 years and that in the control group was 66.4 years. The one-month prehabilitation programme consisted of nutritional counselling, protein supplementation, anxiety reduction and a moderate exercise programme. There was no difference in functional capacity at initial assessment. After one month, the prehabilitation group showed improved post-operative functional recovery.

**Table 2:** Potentially modifiable factors to improve surgical outcome.

Factor	Evidence	Targeted Intervention
Comorbidity	A number of studies have undoubtedly shown that comorbidity is associated with adverse post-operative outcomes. This includes comorbidity measured by number, severity and using a validated comorbidity measurement scale [29,30]	Preoperative optimisation of treatment of all comorbid diseases. Most frequent examples: Heart failure; consider beta blocker and ACE inhibitor treatment, Ischemic heart disease; consider statins, antiplatelet and beta blocker therapy, preoperative percutaneous coronary intervention if unstable disease. Arrhythmias; consider anticoagulant therapy and betablocker therapy or pacemaker implantation. COPD; optimise anti-obstructive therapy. Smoking; cessation program Diabetes mellitus; Optimise glucose-lowering regime Aneamia; assessment of cause. Vitamin B or iron supplements if needed. Preoperative tranfusion. Renal failure; temporary cessation of potential nephrotoxic drugs, adequate fluid balance.
	In a retrospective study of 449 patients aged 65 years and older with invasive and *in situ* breast cancer undergoing surgery, Rocco *et al.* [31] found that 3 or more concomitant diseases and polypharmacy measured pre-operatively, was associated with increased incidence of post-operative complications and therefore worse overall survival.	
	Pei *et al*. [32] retrospectively studied a cohort of 476 patients aged 70 and older with non-small cell lung cancer, undergoing surgical treatment. They found that a number of factors measured pre-operatively increased the risk of post-operative complications: smoking, Charlson Comorbidity Index (CCI) score of 3 or more, duration of surgery greater than 180 min was associated with increased risk of post-operative complications.	
	Musallam *et al*. [33] analysed data for 227,425 patients, 69,229 who had preoperative anaemia. Post-operative mortality was higher in patients with anaemia than those without, regardless of level of anaemia.	
Nutritional status	A study by Takama *et al.* [34] analysed 190 patients aged 75 years and older undergoing gastrectomy for gastric cancer, agreed that greater extent of surgery correlated with increased risk of post-operative complications and further identified that pre-operative malnutrition, was a significant predictor of poor outcome.	Preoperative and postoperative nutritional support [37]
	Jiang *et al.* [35] calculated prognostic nutritional index (PNI) for 385 with gastric cancer. Greater PNI, demonstrating better nutrition, was an independent risk factor for the incidence of postoperative complications and overall survival.	
	A systematic literature review by van Stijn *et al.* [36] analysed the effect of preoperative nutrition on postoperative outcome in elderly general surgical patients. The study included 15 articles using a variety of scoring systems to measure nutrition. Pre-operative weight loss and serum albumin levels were found to predict worse postoperative outcome in this cohort of patients.	
Smoking	Ogawa *et al.* [38] evaluated prognosis after surgery for 727 patients with curative resection for non-small-cell lung cancer. Smoking showed greater risk of postoperative complications and this was more so in the patients aged ≥75 years.	Signposting to “stop smoking” services
	A small study by Gerude *et al.* [39] of 67 patients aged ≥75 years undergoing surgery for head and neck cancer found that smoking was an independent predictor of prolonged length of hospital stay.	
	In the studies by Rocco *et al.* [31] and Pei *et al.* [32] previously mentioned, it was also found that smoking was an independent risk factor for developing post-operative complications.	
Functional reserve— (cardiorespiratory/ physiological function)	Junejo *et al.* [40] analysed 64 patients who had pancreaticoduodenectomy for head of pancreas tumours following cardiopulmonary exercise testing (CPET). They found that raised CPET derived CO2 level predicted early postoperative death and poo long-term survival.	Prehabilitation exercise program
	West *et al.* [41] analysed 136 patients with a median age of 71 who had major colonic surgery, following CPET. They found that measurements significantly lower in patients with complications than without included: O2 uptake, estimated lactate threshold and ventilatory equivalent for CO2.	
Polypharmacy		Cessation of medication that is no longer indicated. Particular focus on medication that increases risk of postoperative complications such as delirium and renal failure.
Cognitive function	Multifactorial intervention [42]	Prevention of delirium through a multifactorial intervention [42] Optimisation of perioperative cerebral perfusion and metabolism; blood pressure control, adequate oxygenation, tranfusion in case of anaemia.
Emotional status	No RCTs done	Psychiatric follow-up, antidepressant therapy.
Social network		Postoperative planning of care, involvment of next of kin.

However, the potential benefits of delaying surgery for prehabilitation must be weighed against the risk of progression of cancer as well as the psychological burden for the older cancer patient who has a tumour needing intervention. If cancer surgery is delayed due to a lack of resources, and in such cases there is no downside to recommending for example physical therapy and training before surgery in order to maximise the patient’s functional capacity. An online survey of European Society of Surgical Oncology (ESSO) and Society of Surgical Oncology (SSO) members conducted by the Surgical Task Force of the International Society of Geriatric Oncology (SIOG) received responses from 251 surgeons regarding opinions on onco-geriatric assessment. Notably, 71% of surgeons stated they would be prepared to delay surgery in favour of prehabilitation, up to 4 weeks, if shown to have better functional recovery [43].

### 2.4. Enhanced Recovery after Surgery (ERAS) Programmes

Enhanced recovery after surgery (ERAS) programmes have been implemented in the UK to optimise recovery for elective surgical patients. They seek to deliver an optimal pathway covering pre-operative, intra-operative and post-operative stages, to focus on optimal recovery and discharge for patients. They cover a range of aspects depending on the type of surgery and can include: nutritional status, mobility, monitoring, analgesia and follow-up [44].

A Cochrane review by Spanjersberg *et al.* [45] investigated the effectiveness of ERAS compared to conventional methods of care in colorectal surgery. A total of four randomised controlled trials (RCTs) analysing a total of 237 patients were presented. The authors found a significant risk reduction for all complications and shorter length of hospital stay in the ERAS group. However, it was noted that the quality of data available was low.

Paton *et al.* [46] conducted a systematic review of the subject. A total of 17 systematic reviews and 12 RCTs for patients on ERAS programmes met the inclusion criteria for analysis in their study. Most of the evidence focused on colorectal surgery and the quality of evidence presented varied greatly, thus making discussion of the results difficult to interpret. The study showed that ERAS programmes may reduce length of hospital stay for colorectal surgery between 0.5–3.5 days compared with standard care. Other surgical specialties showed a greater variation in a reduction in length of stay. In general, it appears that there was a lack of good quality research in this area.

### 2.5. Geriatric Assessment (GA)

Some studies have looked at the role of geriatric assessment in the pre-operative setting of older patients with cancer. A recent systematic review by Feng and colleagues examined the utility of GA components as predictors of adverse outcomes among geriatric patients undergoing major oncologic surgery [47]. The review found six studies that met the inclusion criteria. None of these studies were randomized controlled trials, but prospective observational studies. In summary, functional dependency, fatigue, depression, frailty and cognitive impairment were associated with increased post-operative complications. No GA predictors were identified for post-operative mortality because of a small number of events. Frailty, functional dependency, and depression predicted discharge to a non-home institution. As noted in the paper, the most obvious domains to intervene on in the surgical period are nutritional status and comorbidities. Comorbidities may be optimized, and nutritional supplements may be prescribed. Cognitive dysfunction is not an easy domain to intervene on, but an assessment of cognitive function is necessary in order to obtain informed consent and to identify the risk of post-operative delirium. One could argue that depression is a potentially modifiable factor, but treatment of depression has a longer perspective than the immediate perioperative period. However, depression may be relevant in the preoperative setting if the patient denies treatment due to depression. In summary, it is likely that a multifactorial intervention is the most effective way to optimise older surgical cancer patients. This has been shown for prevention of delirium and for patients with hip fracture [48]. Table 2 specifies domains of a GA and potential interventions (Table 2).

In a systematic review from 2014, authors looked at how pre-operative comprehensive geriatric assessment (CGA) has been used in surgical patients and sought to examine the impact of CGA on post-operative outcomes in older patients undergoing scheduled surgery [49]. This review was not specifically for cancer patients. Two randomised controlled trials and three before-and-after intervention quasi-experimental studies were included. Both the randomised trials showed benefit on post-operative outcomes, while two of the before-and-after studies reported a positive impact on post-operative length of stay and other outcomes. A meta-analysis was not possible due to the heterogeneity of the studies. The authors conclude that pre-operative CGA is likely to have a positive impact on post-operative outcomes in older patients undergoing elective surgery.

### 2.6. Anaesthetic Assessment

Surgery may be a suitable option dependent on characteristics of the cancer but not always for the individual patient. An anaesthetic assessment may be required in order to make these decisions. Although the role of the anaesthesiologist is beyond the scope of this article, they are considered a pivotal member of the MDT in geriatric oncology. High-risk patients should be identified at the pre-operative phase in order to not necessary exclude them from having a surgical procedure, but manage them as appropriate.

The anaesthetist may have a role during surgery for optimisation of haemodynamic status by using pre-specified protocols for the older patient, regarding fluid management. Early studies have suggested these protocols may reduce the risk of complications and length of hospital stay [50,51].

A multicenter randomised observer-blinded trial by Pearse *et al.*, of patients aged ≥50 years undergoing major gastrointestinal surgery, assigned 368 participants to a cardiac-output guided haemodynaemic therapy algorithm for intravenous fluid and inotrope infusion during the peri- and post-operative stage, with 366 having usual care. The authors found that the use of the algorithm did not reduce complications or 30-day mortality. However, inclusion of the data in an updated meta-analysis indicated that the intervention was associated with a reduction in complication rates [52].

## 3. Post-Operative Management

### 3.1. Optimisation of Care in High Risk Patients

If a patient consents for surgery, the decision should be taken regarding their post-operative destination and the need for a higher level of care [53,54]. The European Surgical Outcomes Study [53] showed that surgical mortality for patients ≥16 years undergoing non-cardiac surgery is 3.6% in the UK compared with previous national estimate of 1%–2%. 73% of those who died had never been admitted to critical care wards after surgery. Of those who were admitted to a critical care environment, 43% then died after being transferred to a regular ward. Therefore it is of paramount importance that high risk patients are identified and managed accordingly.

In any surgical population, some patients will recover quicker than others no matter what pre-operative optimisation is put in place. Patients who are most likely to suffer post-operative complications without any intervention may be candidates for support in intensive care or high dependency units. Therefore more intensive care post-operatively can be prepared.

Despite appreciation that age alone should not influence treatment options offered, it appears that increasing age is a risk factor for post-operative complications, including increased morbidity and mortality [55,56], hospital acquired pneumonia [55], necessity for further surgical procedures, bleeding and infection [57], post-operative delirium [56]. This seems to be especially pronounced for emergency procedures. Other factors have been found to predict incidence of post-operative complications in the elderly, some of which have been previously discussed (Table 2) and include surgery for malignancy [29], greater comorbidity [29,30], polypharmacy [31,57], smoking [32], more intensive procedure [32], pre-operative malnutrition [34] and functional dependency [31].

### 3.2. Risk Stratification Tools and Interventions

There are a number of risk predictor tools which may be of use in stratifying patients at risk of poor postoperative outcome. Currently used in clinical practice are the American Society of Anesthesiologists (ASA) scoring system, Goldman Cardiac Risk Index (CRI), Acute Physiological and Chronic Health Evaluation (APACHE) score and Physiological and Operative Severity Score for EnUmeration of Mortality and Morbidity (POSSUM). The scores measure a variety of risk factors and should be used in combination with clinical findings to guide decision making in regards to who should have a higher level of care.

There are a number of tools with the potential to be used in the post-operative phase, in order to monitor the at risk patient for signs of deterioration. The Vascular Events in Noncardiac Surgery Patients (VISION) study [24] is an international, prospective cohort study of 15065 patients aged ≥45 years undergoing in-patient noncardiac surgery. Myocardial injury due to ischaemia was measured by serial troponin levels. The study found that an elevated troponin after noncardiac surgery, irrespective of the presence of an ischaemia feature, predicted 30 day mortality.

### 3.3. Integrated Care by the Geriatrician

Rehabilitation after surgery starts in the immediate post-operative phase. The geriatrician can provide his or her expertise in working in interdisciplinary teams, with all team members contributing their part in the patient’s recovery. The geriatrician will take part in the daily assessment of patients, and provide typical geriatric medicine services such as optimisation of patients’ medication, pain relief, reinforce early mobilisation, prevention of delirium and other medical complications as well as identification and treatment of medical complications that arise.

Post-operative complications in elderly patients have been shown to reduce long-term survival and increase risk of readmissions [58,59]. The interdisciplinary team will work to prevent complications through such measures as early per oral nutrition to promote wound healing and reduce risk of infections, early mobilisation and chest physiotherapy to help prevent pneumonias and functional decline, avoidance of indwelling catheters to reduce the risk of infections and adjustments of medication to avoid post-operative hypotension and renal failure.

If complications occur despite preventive measures, they should be diagnosed and treated as swiftly as possible, as the older patient in general has less reserve capacity, and will start to deteriorate sooner than a younger patient. However, diagnosing complications is often not straight-forward in the frail older patient. They will generally present with diffuse symptoms of acute illness, often referred to as “geriatric syndromes” [60]. Typical examples are delirium, eating difficulties, incontinence and falls. The occurrence of such symptoms should always raise the suspicion that a complication has occurred, and lead to adequate diagnostic and therapeutic measures.

The discharge planning for each older patient should ideally start pre-operatively, when deciding on surgery. Transitions between different levels of care for older patients with many comorbid diseases and polypharmacy is an area where complications often occur. The geriatrician has wide experience in this field, and will start discharge planning in accordance with the needs of the patient and the family as early as possible to reduce the risk of adverse events.

There is still no high-quality evidence for this integrated geriatric-surgical approach in geriatric cancer surgery. There are small, non-randomised studies that have shown promising results on length of stay and in regaining pre-operative functional status after colorectal surgery [61]. There is, however, growing evidence for this integrated model from other surgical areas, in particular in orthopaedic surgery, where systematic reviews have shown beneficial effects of ortho-geriatric care on delirium, occurrence of complications, and mortality [57,62]. In a recent randomised trial of a geriatric liaison intervention to prevent post-operative delirium in frail elderly cancer patients, however, the intervention was not proven to be effective [63]. The trial included 260 patients with a mean age of 77 years who were operated for a solid tumour.

The organisation of geriatric oncology varies considerably internationally, and even within Europe [63]. In other fields of geriatric research the best results for treatment of frail older patients with a variety of conditions are obtained when patients are treated as inpatients in a geriatric ward. A recent randomised controlled trial of patients with hip fracture found that patients had better functional outcomes when being admitted to the geriatric ward instead of the orthopaedic ward. The orthopaedic surgeons performed surgery and were consultants on the geriatric ward for patients in the intervention group [48]. However, in clinical practice this model of care is often not possible due to a lack of resources. In many cases, geriatricians serve as liaisons on other medical wards, even though this organisation is less efficient [64].

## 4. Conclusions

Cancer surgery in the elderly patient has different aims and implications when compared to the younger population (Table 3). Older patients are more likely to suffer from comorbidity and frailty. As a result, it is crucial that the correct surgical procedure is identified and that the patient is medically optimised in the pre- and post-operative setting. There is growing evidence for the use of prehabilitation to improve nutritional status and general physical fitness in elderly patients undergoing cancer surgery. In some specialties, for example colorectal surgery, it is now commonplace to use an ERAS programme to decrease the incidence of complications.

**Table 3:** Comparison of key points from surgical *versus* geriatric perspective.

	Surgical Key Points	Geriatric Key Points
Selection for surgery	Intent of surgery e.g., curative *versus* palliative Extent of surgical procedure required Alternative non-operative treatment available	Remaining life expectancy Frailty Cognitive function Patient preferences
Pre-operative assessment	Concept of prehabilitation Optimisation of comorbidities Identification of post-operative intensive care needs	Functional status Physical performance Comorbidities Cognitive function Nutritional status Emotional status Social network
Post-operative management	Optimisation of care, including intensive care if necessary, in high risk patients Concept of early rehabilitation	Managing complications Prevent delirium Early rehabilitation
Potential models of care	Multidisciplinary team, including surgeon and geriatrician, performs geriatric assessment and intervention Dedicated clinic with surgeon and geriatrician in attendance Geriatric liaison performs geriatric assessment and intervention	

Where appropriate, consideration of higher level of care post-operatively should be discussed. Risk factors for poor operative outcome should be identified early and include increasing age, comorbidity, functional dependency, cognitive dysfunction, frailty and intensity of procedure. This will allow for appropriate measures to be put in place pre-operatively as well as optimised post-operative care.

We have identified the roles of both the surgeon and geriatrician during the whole journey of the elderly patient with cancer and explicitly identified the geriatrician as being a key member in providing care beyond which can be offered by the surgeon alone. Both surgeons and geriatricians are used to working in a MDT to make individual treatment plans, however this is not currently done in unison in the setting of geriatric oncology. This highlights the need for a potential provision of parallel services from both of these teams to aid overall management of such patients.

Several potential models exist to improve current care. One suggestion is the introduction of a screening GA tool implemented by surgeons, which may trigger a geriatric referral. Another potential model of care in the older patient with cancer would be to routinely incorporate a geriatrician as a core member of the MDT discussion. A third potential model of care would be to have a dedicated pre-operative clinic for the older adult with cancer, where the operating surgeon and geriatrician are both present at the initial consultation.

In the post-operative phase, the geriatrician can provide daily assessments of the patients, optimise medications, reinforce early mobilization, work to prevent medical complications, identify and treat those complications that arise, and start discharge planning as early as possible to ensure less complicated transitions between different levels of care.
